# Ibrutinib Monotherapy as Bridge-to-Transplant for Relapsed/Refractory Primary Oculo-Cerebral Lymphoma

**DOI:** 10.3390/jcm10194483

**Published:** 2021-09-29

**Authors:** Dalma Deak-Mihaly, Sabina Iluta, Sergiu Pasca, Ciprian Jitaru, Andrei Roman, Alexandra Andries, Monica Padurariu-Covit, Bobe Petrushev, Anca Vasilache, Anca Bojan, Mihnea Zdrenghea, Angela Dascalescu, Ion Antohe, Anca Colita, Andrei Colita, Delia Dima, Alina Tanase, Ciprian Tomuleasa

**Affiliations:** 1 Department of Hematology, Iuliu Hatieganu University of Medicine and Pharmacy, 400005 Cluj Napoca, Romania; dalmadeak26@yahoo.com (D.D.-M.); iluta.sabina@yahoo.com (S.I.); pasca.sergiu123@gmail.com (S.P.); ancasbojan@yahoo.ca (A.B.); m.zdrenghea@yahoo.com (M.Z.); 2 Medfuture Research Center for Advanced Medicine, Iuliu Hatieganu University of Medicine and Pharmacy, 400124 Cluj Napoca, Romania; bobe.petrushev@gmail.com; 3 Department of Hematology, Ion Chiricuta Clinical Cancer Center, 400125 Cluj Napoca, Romania; Ciprianjitaru.jitaru@gmail.com (C.J.); ancavasilace@gmail.com (A.V.); deli_dima@yahoo.com (D.D.); 4 Department of Radiology, Iuliu Hatieganu University of Medicine and Pharmacy, 400124 Cluj Napoca, Romania; andrei.roman678@gmail.com; 5 Department of Radiology, Ion Chiricuta Clinical Cancer Center, 400124 Cluj Napoca, Romania; sanda_andries@yahoo.com; 6 Department of Internal Medicine, Sf. Apostol Andrei Emergency County Hospital, 800366 Galati, Romania; monica_monica1406@yahoo.com; 7 Department of Hematology, Grigore T. Popa University of Medicine and Pharmacy, 700115 Iasi, Romania; angdascalescu@yahoo.com (A.D.); antohe_ion@yahoo.com (I.A.); 8 Department of Stem Cell Transplantation, Regional Institute of Oncology, 700115 Iasi, Romania; 9 Department of Stem Cell Transplantation, Fundeni Clinical Institute, 420003 Bucharest, Romania; ancacolita@yahoo.com (A.C.); alinadanielatanase@yahoo.com (A.T.); 10 Department of Pediatrics, Carol Davila University of Medicine and Pharmacy, 420003 Bucharest, Romania; 11 Department of Hematology, Coltea Hospital, 420003 Bucharest, Romania; andreicolita@yahoo.com; 12 Department of Hematology, Carol Davila University of Medicine and Pharmacy, 420003 Bucharest, Romania

**Keywords:** primary central nervous system lymphoma, relapsed/refractory, ibrutinib, bridge-to-transplant

## Abstract

Introduction. Primary central nervous system lymphoma is an uncommon form of extranodal non-Hodgkin’s lymphoma, with increasing incidence, a relatively aggressive course and a poor 5-year survival. Because of its localization, the therapeutic compounds used in this disease must be able to pass through the blood-brain barrier. Chemotherapy regimens based on high-dose methotrexate are currently the standard of care for all patients who can tolerate such drugs. Autologous stem cell transplantation is indicated for malignant lymphomas in the relapsed/refractory setting. Methods. Three patients, with a median age of 60 years, range 53–64, were diagnosed with primary CNS lymphoma, and treated with ibrutinib monotherapy in the Department of Hematology, Ion Chiricuta Clinical Cancer Center, Cluj-Napoca, Romania, between September 2018 and November 2020 All the patients were relapsed–refractory following high-dose methotrexate chemotherapy. We present our experience using ibrutinib monotherapy-based treatment as a bridge-to-transplant option on a single-center case series and a review of the literature in this field. Results. Two of the patients were given ibrutinib as a second line therapy, both achieving complete remission and being eligible for an autologous stem cell transplantation. The third patient achieved a short remission using six cycles of systemic chemotherapy, but was started on ibrutinib monotherapy, with limited results. Conclusion. Our data is limited, and these results should be confirmed by multicentric clinical trials and should be regarded as a single-center case series, with all its limitations. Still, it brings forward a new therapeutic option for this rare subtype of malignant lymphomas, which if left untreated has a dismal prognosis.

## 1. Introduction

Primary central nervous system lymphoma (PCNSL) is a rare and aggressive form of extranodal non-Hodgkin’s lymphoma, with increasing incidence and a poor 5-year survival [1,2]. Because of its localization, the therapeutic compounds used in this disease must pass through the blood-brain barrier. In the late 1990s, the radio-chemotherapy was the gold standard, but because of the high risk of neurotoxicity associated with whole brain radiotherapy (WBRT), physicians in recent times have recently preferred a radiotherapy-free approach. Thus, the cornerstone of therapy remains high-dose methotrexate with or without other chemotherapeutic agents that have activity against PCNSL [1,2,3,4]. As the malignant clone can develop resistance to methotrexate, or the patients can develop severe adverse events, further alternatives are thus required. A promising agent is represented by ibrutinib [5,6]. This molecule might be a good therapeutic choice as it has been shown that PCNSL is dependent on B-cell receptor (BCR) signaling and thus has a sensitivity to ibrutinib.

Based on this data, ibrutinib monotherapy was investigated in a French phase II clinical trial enrolling 52 patients, which reported that ibrutinib monotherapy achieved disease-control in 70% of patients [7]. Recently, the Australasian Lymphoma Alliance/MD Anderson Cancer Center experience reported an interim analysis on 33 patients who received ibrutinib alone or with other therapies, with an objective response rate of 58% [8]. Still, ibrutinib monotherapy has limited efficacy because of the aggressiveness of the disease and resistance to therapy, both in conventional chemotherapy and targeted therapies. Autologous stem cell transplantation is indicated for malignant lymphomas in the relapsed/refractory setting [9,10,11,12]. In the current manuscript, we present a single-center experience of using ibrutinib monotherapy as a bridge-to-transplant option for relapsed/refractory (R/R) PCNSL, with promising results.

## 2. Patient Information

We present our experience using ibrutinib monotherapy-based treatment as a bridge-to-transplant option on a single-center case series. Three patients, with a median age of 60 years, range 53–64, were diagnosed with primary CNS lymphoma, and treated with ibrutinib monotherapy in the Department of Hematology, Ion Chiricuta Clinical Cancer Center, Cluj-Napoca, Romania, between September 2018 and November 2020 (Table 1).

All the patients were relapsed–refractory following high-dose methotrexate chemotherapy. Two of the patients were given ibrutinib as a second-line therapy, both achieving complete remission and being eligible for an autologous stem cell transplantation. The third patient achieved a short remission using six cycles of systemic chemotherapy (rituximab plus ICE protocol) [9], but was started on ibrutinib monotherapy, with limited results (Table 1). Further, the detailed case presentations are presented as follows.

**Figure 1:**
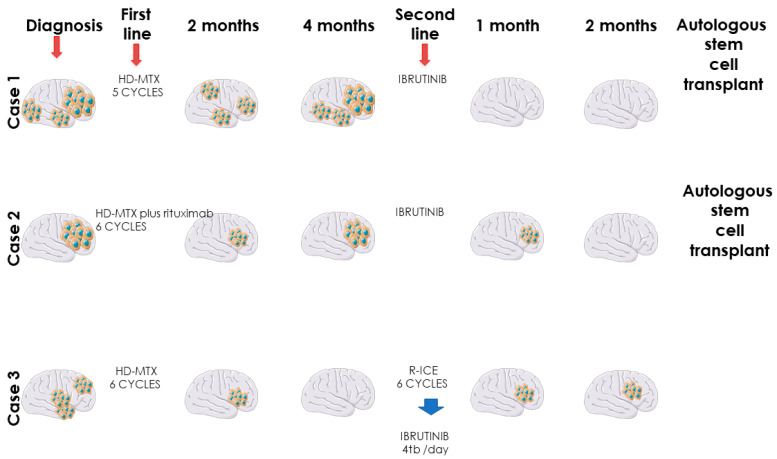
Overall clinical evolution of primary CNS lymphomas treated with ibrutinib.

**Figure 2:**
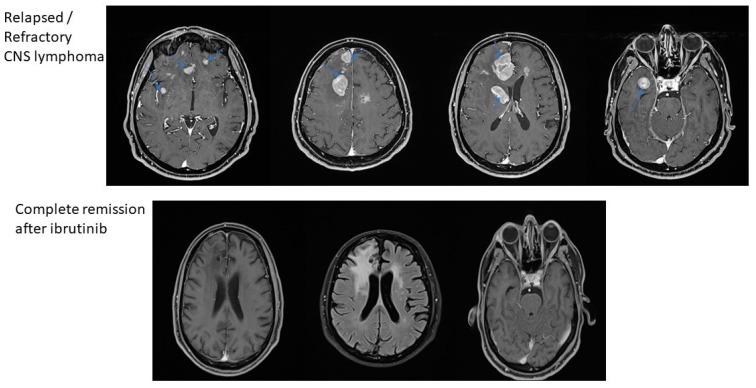
MRI-based remission of CNS lymphomas treated with ibrutinib.

The first case is a 63-year-old female with chronic gastritis, chronic liver disease and gastro-esophageal reflux disease, and diagnosed with PCNSL. The clinical presentation was represented by diffuse headaches, diplopia, and equilibrium alterations. At the clinical examination, the general status was slightly altered with no adenopathy or alterations of the pulmonary or cardiovascular systems. Due to the important increase in the intensity of the headaches, the patient was investigated in a neurosurgery service using a magnetic resonance imaging (MRI), which revealed multiple gadofilic masses, with moderate perilesional edema and moderate diffusion restriction. These masses were situated at the right parietal posterior region, right thalamic region, in both temporal lobes and in the right frontal lobe. A biopsy was then performed, which, under histopathologic examination revealed a primary central nervous system lymphoma (PCNSL). The patient was started on a regimen of high dose methotrexate which was kept for five cycles, without the patient achieving complete remission. The adverse events associated with this treatment have been represented by mild mucositis and pulmonary and pharyngeal infections, which have been treated with antibiotic therapy. After these cycles, the patient was started on 560 mg/day of ibrutinib. After this, she presented a complete response, and she is currently eligible for autologous stem cell transplantation (ASCT) in a secondary transplant center (Figure 1 and Figure 2).

The second case is a 53-year-old female patient, without relevant pathological history, diagnosed with primary ocular diffuse large B cell lymphoma, stage IE (right eye), at the Jules Bordet Institute in Brussels, Belgium. The clinical presentation showed weight loss and granulomatous bilateral anterior uveitis, manifested with altered visual acuity. At diagnosis, the MYD88 L265P mutation was detected, which is associated with non-Hodgkin’s lymphomas with poor overall survival (OS). The patient was treated with intraocular administration of methotrexate (MTX) and rituximab, associated with MATRIX regimen (without thiotepa), administered for 6 cycles. Unfortunately, after 2 month of finishing chemotherapy, the lymphoma affected the patient’s left eye. The patient presented with severe and fast cognitive degradation and after a second biopsy, diagnosis of PCNSL was confirmed, it having infiltrated both frontal lobes. The second-line therapy was ibrutinib (560 mg/day), a treatment which continued for 4 months. The control MRI performed in July and September of the same year depicted demyelination of the white substance in frontal left lobe, but with remission of the CNS tumoral masses. The patient still presents headache, but PET scan made in September reveals complete metabolic remission and she is currently eligible for autologous stem cell transplantation (ASCT) in a secondary transplant center (Figure 1).

The third case is a 64-year-old male patient, known with multiple cardiovascular diseases, diabetes, and dyslipidemia, diagnosed with PCNSL. The patient presented memory disorder and alteration of the mental status. The MRI showed bilateral frontal lobe invasion of the tumoral masses. The patient started high dose methotrexate regimen and, after six cycles, the control MRI describes right frontal post craniotomy status with adjacent gliosis and residual edema, without signs of evolution. After complete remission, two cycles of high-dose cytarabine were administered as consolidation therapy. After one year, the patient presented balance disorders, headache, diplopia, weight loss, nausea, and vomiting. The emergency MRI depicted a right temporo-parietal tumor, right occipital ventricular tumor, with 32/23 mm diameter, associated with edema and mass effect. As second-line treatment, R-ICE chemotherapy regimen (rituximab, ifosfamide, carboplatin and etoposide) was administered, six cycles long. The control MRI following therapy depicted bilateral fronto-parietal juxtacentimetric demyelinated lesions on a background of leukoaraiosis of the frontal horns, and without tumoral masses. At this point, the patient refused an ASCT. Four months later, the patient presented intense headache, fatigue, nausea, vomiting and ataxia. The MRI showed relapsed masses in the frontal lobe. The third line therapy was ibrutinib (560 mg/day), but after two months, the control MRI showed progressive disease and altered clinical status (Figure 1).

## 3. Discussions

Primary central nervous system lymphoma (PCNSL) is a highly aggressive non-Hodgkin’s lymphoma type arising in the central nervous system (CNS), widely involving the brain parenchyma, spinal cord, leptomeninges, eyes, cranial nerves and/or meninges [13,14]. These accounts for 3.3% of intracranial tumors, and because of the low incidence and limited drug options, efficient treatment is limited. Overall survival (OS) rate in patients with PCNSL and long-term survival is much lower than the same histological type of lymphoma involving peripheral lymphoid organs [15,16,17]. PCNSL appears frequently among elderly or immunocompromised (HIV/AIDS-acquired immune deficiency syndrome, organ transplant, immunosuppressive agents) patients. The incidence is slightly higher in male patients, and in Caucasians. The median age at diagnosis is 60 years in immunocompetent patients, and 35 years in immunocompromised ones. PCNSL incidence increased slightly from 1960 to 1995. After 2000, the incidence stayed permanent among patients under 65 years old, because of decreased incidence of AIDS and the development of specific treatment of AIDS. However, among patients more than 65 years, the incidence is still rising. The most common histologic subtypes of PCNSLs are DLBCL (90–95%) and, rarely, Burkitt, low-grade, or T-cell lymphoma. Patients are complaining about mental status or behavior changes, headache, nausea, vomiting and seizures, but most often about focal neurologic deficits (around 70%). The lymphoma is often defined as a single brain lesion, usually supratentorial (87%), with frontoparietal lobes involvement. Although it has a favorable response to chemotherapy and radiotherapy, the survival is usually inferior to other lymphomas.

Radiotherapy has been used for decades as the treatment of PCNSL, but its role has been diminishing recently. Clinical data has shown that can lead to serious neurotoxicity, such as cognition, memory and other functions impairment, brain atrophy, leukoencephalopathy, endocrine disorders and even dementia [18,19,20]. A growing number of clinical trials have shown the efficacy of several treatment strategies, but high-dose methotrexate still represents a milestone in the chemotherapy on PCNSL because of it crosses the blood-brain barrier (BBB). The key role of methotrexate is confirmed by the fact that those patients who cannot tolerate high-dose methotrexate have a poor prognosis. Methotrexate is a folate antagonist that interrupts DNA synthesis and is now the standard-of-treatment for PCNSL. To achieve therapeutic concentrations of MTX in the central nervous system, high doses are required (at least 1.5 g/m^2^). As the steady-state blood: CSF ratio is about 30:1, doses less than 3 g/m^2^ are too low to achieve anti-cancer effects in the cerebrospinal fluid (1 μmol/L). A three-hour infusion of 3 g/m^2^ of MTX achieves higher CSF levels than slower infusions of higher concentration of MTX. Data regarding surgical treatment of PCNSLs is scarce. Previously published data agree that resection is discouraged [21,22,23]. 

High-dose chemotherapy (HDC) with autologous stem cell transplantation (ASCT) has been investigated for post-remission consolidation and for relapsed disease, with promising results [11,12,24,25,26,27,28]. HD-MTX–based induction regimens, followed by ASCT, with various conditioning regimens, including BEAM (carmustine, etoposide, cytarabine, melphalan) [29], thiotepa, busulfan [30,31], TBC (thiotepa, busulfan, cyclophosphamide) [32,33,34] and thiotepa, carmustine [35,36,37,38] have been evaluated. The data thus far, however, have been limited to single-arm phase II-III trials, and several important questions remain, including the best candidates for such therapy, timing of its use, possible toxicities, and optimal conditioning regimen. Young et al. support the use of high-dose chemotherapy followed by ASCT with CNS-directed conditioning as consolidative therapy for PCNSL with long-term efficacy and manageable side effects. Patients with PCNSL who had undergone ASCT in the first complete remission (CR1) appeared to have a better survival benefit compared with those patients who had undergone ASCT after CR1. Data for SCNSL and in the relapse setting also appear encouraging. Longer follow-up and studies based on comparison with other consolidative therapies will provide further insight and should be reported soon.

The French Oculo-cerebral lymphoma network (LOC) reported that in a large cohort of 1000 patients treated in 32 centers, that first-line treatment was high-dose MTX-based chemotherapy in 92% of cases, with an increasing use of rituximab over time (66%). Patients younger than 60 years received consolidation treatment in 77% of cases, consisting of WBRT (54%) or high-dose chemotherapy with ASCT (23%). Among patients older than 60 years, WBRT and ASCT consolidation were administered in only 9% and 2%, respectively. The CR rate to initial chemotherapy was 50%. Median PFS was 10.5 months. For relapse, second-line chemotherapy, ASCT, radiotherapy, and palliative care were offered to 55%, 17%, 10%, and 18% of patients. The median, 2-year, and 5-year overall survival were 25.3 months, 51%, and 38%, respectively. Age, sex, and response to induction CT were independent prognostic factors in a multivariate analysis [39]. The same LOC reported a proof-of-concept phase II clinical trial using ibrutinib monotherapy for relapsed/refractory PCNSL and after two months of treatment, the disease control was 70% in evaluable patients and 62% in the intent-to-treat analysis, including complete responses in 19% of patients, partial responses in 33% and stable diseases in 10% of cases. With a median follow-up of 25.7 months, the median progression-free and OS were 4.8 months and 19.2 months, respectively [7]. 

In recent decades, immunotherapies, targeted therapies, adoptive cell transfer, monoclonal antibodies, and vaccine treatments, have become efficient and highly specific treatments to attack tumor cells by activating the patient’s immune system. Immunotherapy can specifically mark the malignant cells and their environment with less cytotoxicity and fewer side effects. The discovery and development of small molecule cancer drugs has been revolutionized the therapy of cancer in the last few decades. Instead of the use of the one-size-fits-all approach that characterizes the cytotoxic chemotherapy, small molecules have made possible a personalized medicine strategy that focuses on the targeted drugs that exploit the genetic addictions, dependencies, and vulnerabilities of cancer cells. As a result, it led to an increasing number of successful therapies that have favorably influenced the lives of numerous patients diagnosed with cancer. Hematological malignancies can be characterized as a “miscommunication” disease, as the initiation and the further progression of the disease is based on over activations of numerous extrinsic and intracellular signaling pathways. Small molecule drugs have been successfully used to target the extracellular, cell surface ligand-binding receptors and intracellular proteins, which play an essential role in transducing downstream signaling for the cell growth and metastatic promotion. These compounds are usually ≤ 500 Da in size and are often administered orally. Their small size also allows them to translocate through the plasma membrane and interact with the cytoplasmic domain of cell-surface receptors and intracellular signaling molecules. In principle, small molecule compounds can be developed to target any portion of a molecule, regardless of the target’s cellular location. Most of these small molecules suppress crucial cancer targets such as serine/threonine/tyrosine kinases, matrix metalloproteinases (MMPs), heat shock proteins (HSPs), proteasome and other proteins playing a role in signal transduction pathways.

Although a various number of small molecules are successfully utilized in the clinic, the appearance of drug-resistant variants of cancer represents a significant problem and raises the necessity of development of novel signaling molecules to effectively treat resistant cancers in the clinic.

As a first-generation Bruton-kinase (BTK) inhibitor, ibrutinib was rapidly approved by the US Food and Drug Administration (FDA), firstly for the treatment of chronic lymphocytic leukemia (CLL)/small lymphocytic lymphoma (SLL) and mantle-cell lymphoma (MCL) in 2013 and 2014. Since then, it was continuously approved for use as single agent in patients with other B-cell lymphomas. 

The B cell receptor (BCR) pathway regulates multiple cellular processes essential for the functioning and survival of both normal and malignant B cells. In B cell malignancies, BCR signaling plays a critical role in the pathogenesis of disease. The BCR pathway is responsible for the phosphorylation of numerous protein tyrosine kinases (PTKs), including Lyn, Syk, and Bruton’s tyrosine kinase (Btk). These kinases are constitutively active and over-expressed in malignant lymphocytes, leading to uncontrolled proliferation and survival of malignant B cells. Thus, there has been rapid clinical development of inhibitors targeting these PTKs. Among the many PTKs involved in BCR signaling, Btk, a tyrosine kinase member of the Tec kinase family, is a distinctive therapeutic target. Following BCR activation, Btk is activated by other PTKs, such as Lyn and Syk, resulting in activation of downstream transcription factors necessary for B cell proliferation and differentiation. Ibrutinib is an irreversible small-molecule inhibitor of Btk. It forms a covalent bond with a cysteine residue (CYS-481) at the active site of Btk, leading to inhibition of Btk enzymatic activity. Ibrutinib inhibits the full activation of Btk by inhibiting its autophosphorylation at Tyr-223. This inhibition prevents downstream activation of the BCR pathway and subsequently blocks cell growth, proliferation, and survival of malignant B cells.

Ibrutinib, as monotherapy, has been enough to induce a satisfying response rate as well as offering for these patients a better quality of life. Recently, ibrutinib has been proposed as a promising target drug for diffuse large B-cell lymphoma (DLBCL). The two major subtypes of DLBCL are ABC (activated B cell-like) and GCB (germinal center B cell-like), which are induced by different mechanisms. As it is known, the DLBCL is dependent on BCR signaling, thus pathways activated by this receptor might be targeted to obtain a better response in some DLBCL subtypes, including PCNSL [40]. Important downstream signaling molecules activated by BCR are represented by BTK and PI3K, thus, therapeutic options targeting BTK and PI3K have been studied for DLBCL [41]. Ibrutinib, a BTK inhibitor, has been proposed for the treatment of this disease. The inspiration for BTK inhibitors can be thought as being the pharmacological simulation of Bruton’s agammaglobulinemia, an X-linked disease which produces, in the case of men, agammaglobulinemia and in the case of women the only lymphocytes that reach development [42]. Because of this, it has been considered for a long time that BTK is of utmost importance for B-cell development and through extrapolation for B-cell malignancies. One of the most important compounds used for inhibiting BTK is considered to be ibrutinib which binds to C481 of the active site of BTK restraining it’s activity [43]. Due to this, it was first used in chronic lymphocytic leukemia (CLL) with good results, which made it a standard therapy for this disease [44]. After this, it has been applied in other B-cell malignancies such as mantle cell lymphoma (MCL) [45], with other clinical trials further extending the use of ibrutinib to other B-cell diseases. Since ibrutinib r more and more diseases, other BTK inhibitors have been added to the arsenal. For example, acalabrutinib recently received FDA approval to be used in CLL as an alternative to ibrutinib for patients that do not tolerate ibrutinib [46]. Thus, this could also be extended to cases of PCNSL that do not tolerate ibrutinib as an off-label use of acalabrutinib. Another issue that could arise in the case of BTK inhibitor therapy is represented by resistance to therapy with some important mutations being localized in *CD79B* and *CARD11* genes, these being important as they are part of the BCR downstream signaling. More than this, *CD79B* mutations were shown to be associated with an increase in the PI3K/mTOR signaling and with the inhibition of PI3K/mTOR being shown to synergize with ibrutinib in the case of PCNSL mutated cells harboring *CD79B* mutations. Due to this, it can be hypothesized that, in the case of PCNSL acquiring ibrutinib resistance through *CD79B* mutations, the addition of PI3K/mTOR inhibitors could reestablish response to therapy [5].

In phase 2 clinical study, ibrutinib monotherapy produced a response rate in 37% of ABC cases, in contrast with the GCB-DLBCL cases, where the response rate was only 5%. Moreover, the best response rate was observed in tumors with MYD88 and BCR-associated protein CD79B mutations. This explains the ibrutinib success in lymphoplasmacytic lymphoma and Waldenstrom’s macroglobulinemia. At this moment, NCCN has recommended ibrutinib as the second-line and subsequent therapy for transplant-ineligible patients with non-germinal center B-cell lymphomas and optional regimens for patients with PCNSL with R/R disease, as is the case of our patients.

Primary or secondary CNS lymphoma is a rare subtype of extranodal lymphoma, with a very poor prognosis and a median survival of only 2 months without additional treatments. Most of the PCNSL are DLBCL, and high-dose MTX-based regimens are considered standard for newly diagnosed PCNSL. Despite of response rates with first line therapy, more than half of initial responders relapse. Moreover, about 25% of patients have refractory disease to the treatment. The optimal treatment for R/R PCNSL is poorly defined, not to mention their serious side effects. Ibrutinib can provide extended disease control for several patients or as a part of a bridging strategy prior to transplant. The efficacy may be greater in cases of lower tumor burden and in patients previously treated with AHSCT.

In clinical practice, we must mention the adverse events of ibrutinib, which is represented most often by hemorrhagic events, but other grade 3 adverse events have been reported, such as arterial hypertension, pneumonia and neutropenia [5]. Although ibrutinib is generally well tolerated, there are patients that are forced to discontinue ibrutinib therapy because of the acquired side effects. For these patients, acalabrutinib could be a treatment option, as mentioned before [46].

Our experience is limited to a single-center study, with three patients, with comparable results, therapy with ibrutinib as a single-agent be followed by ASCT. The results are excellent, with patients having a better clinical outcome if they are treated with ibrutinib after the first relapse and transplanted after CR1. Still, more data on large, randomized trials is necessary before we can draw any conclusive results.

## 4. Conclusions

Our data is limited, and these results should be confirmed or infirmed by multicentric clinical trials and should be regarded as a single-center case series, with all its limitations. Still, it brings forward a new therapeutic option for this rare subtype of malignant lymphoma, which even when treated has a dismal prognosis. Our initial conclusion is that an autologous stem cell transplantation is compulsory following remission in PCNSL. Ibrutinib can offer a bridge-to-transplant for relapsed–refractory patients. 

## Figures and Tables

**Table 1:** Clinical evolution of the patients.

	Disease Status	Sex	Age at Diagnosis	First Ct/MRI Scan	First line of Treatment and Number of Cycles	Evolution at 2 Months	Evolution at 4 Months	Second Line Treatment	Evolution after Second Line— After 1 Month	Evolution after Second Line— After 2 Months
Case 1	Complete remission	F	63	Right temporal lobe 14/17 mm, right frontal lobe 8 mm, right region of occipital lobe 17/17/22 mm	High-dose methotrexate—5 cycles	Right frontal lobe 7 mm, 2 new lesions: right temporal lobe 10 mm and right parietal lobe 8 mm, without other gadofilic masses	Right frontal lobe 27 mm, right temporal lobe 10 mm, new lesion in right temporal lobe 14 mm.	Ibrutinib monotherapy	No visible lesion	No visible lesion
Case 2	Complete remission	F	53	Left frontal lobe 70/40/30 mm	High-dose methotrexate and systemic Rituximab—6 cycles	Stationary disease	Left frontal lobe 50/30/20 mm.	Ibrutinib monotherapy	Left frontal lobe 32.6 mm metabolically inactve	No visible lesion
Case 3	Refractory	M	64	Bilateral and insular frontal lobe, superior right frontal region, right basal nuclei and right temporal lobe	High-dose methotrexate—6 cycles	Stationary disease	No lesions at MRI scan.	R-ICE 6 cycles, then second remission was achieved, but after 1 month second relapse: right frontal lobe lesion 20/30 mm. Ibrutinib monotherapy as 3rd line treatment	Stationary disease	Stationary disease

## Data Availability

The data that support the findings of the study are available from the corresponding author upon reasonable request.
